# C-Reactive Protein to Albumin Ratio Predicts Early Mortality in Hospitalized Older Patients, Independent of the Admission Diagnosis

**DOI:** 10.3390/nu17121984

**Published:** 2025-06-12

**Authors:** Cristiano Capurso, Aurelio Lo Buglio, Francesco Bellanti, Gaetano Serviddio

**Affiliations:** Department of Medical and Surgical Sciences, University of Foggia, Viale Luigi Pinto 1, 71122 Foggia, Italy; aurelio.lobuglio@unifg.it (A.L.B.); francesco.bellanti@unifg.it (F.B.); gaetano.serviddio@unifg.it (G.S.)

**Keywords:** malnutrition, inflammation elderly, mortality, albumin, C reactive protein, albumin, PCR/Alb ratio

## Abstract

**Background:** Malnutrition and systemic inflammation are prevalent among older hospitalized patients and are associated with increased morbidity and mortality. The C-reactive protein to albumin (CRP/Alb) ratio reflects inflammatory and nutritional status and may serve as a useful prognostic biomarker. **Objective:** To evaluate the prognostic value of the CRP/Alb ratio in predicting early in-hospital mortality in a large cohort of elderly patients, independent of the admission diagnosis. **Methods:** This retrospective observational study examined the clinical data and serum values of serum C-reactive protein (CRP), albumin, and the CRP/Alb ratio, detected at the time of admission, in a cohort of 2780 patients over sixty-five admitted to the Internal Medicine and Aging Department of the “Policlinico Riuniti” University Hospital Trust in Foggia, between 2019 and 2024. The predictive power of the CRP/Alb ratio for 7- and 30-day hospital mortality was evaluated by ROC curve analysis, Cox regression, and Kaplan–Meier survival analysis. **Results:** In total, 444 patients died (16%) during their in-hospital stay. The CRP/Alb ratio was significantly higher among deceased subjects (*p* < 0.001) than in non-deceased patients. The CRP/Alb ratio was strongly associated with mortality, particularly during the first 7 days from admission (AUC = 0.888). A CRP/Alb ratio >8 was an independent and significant predictor of mortality within 30 days (HR = 3.82, 95% CI: 2.91–5.01), but particularly within the first 7 days from hospitalization (HR = 10.17, 95% CI: 6.05–17.08). Similar results were observed among re-hospitalized patients. **Conclusions:** The CRP/Alb ratio is a significant and independent predictor of early in-hospital mortality in elderly patients, regardless of admission diagnosis. A threshold value >8 identifies individuals at high risk, particularly within the first week of hospitalization. This simple, cost-effective biomarker may support early risk stratification and guide targeted interventions in geriatric care.

## 1. Introduction

Population aging is a now evident global phenomenon and one of the most significant challenges of our century. According to WHO data, the number of people aged 65 and over is increasing, both in absolute numbers and as a percentage of the population. This increase is occurring at a rate unprecedented in history and is expected to accelerate worldwide in the coming decades. In 2019, the number of people aged 65 and over was 1 billion. By 2030, 1 in 6 people in the world will be aged 65 or over. By 2050, the global population of people aged 65 and over will double (2.1 billion). The number of people aged 80 and over is expected to triple between 2020 and 2050, reaching 426 million [1].

This will consequently lead to an increase in the number of people affected by numerous chronic diseases, or by the well-known geriatric syndrome of frailty. Frailty is defined as a condition of increased vulnerability, or reduced resilience, following even a mild pathogenic event, with the consequent increase in the risk of adverse outcomes, including falls, delirium and disability [2,3,4,5,6,7], in addition to a significant increase in the care burden and expenses due to numerous and repeated hospital admissions [8,9,10].

Elderly patients, who are admitted to acute care units, already suffer from chronic diseases at the time of admission, with signs and symptoms of physical and cognitive deterioration, malnutrition, and complex home therapies characterized by the intake of numerous drugs [11]. Concerning malnutrition, we commonly refer to the depletion of protein reserves due to reduced intake, with wasting and a significant reduction in body weight, or due to a catabolic state triggered by systemic inflammation secondary to the ongoing concomitant disease [12,13].

The prevalence of malnutrition among hospitalized patients varies between 15% and 70%, depending on the case history and the conditions of hospitalization [14,15,16,17,18,19,20,21]. Several factors contribute to malnutrition, such as the presence of pre-existing chronic diseases, pathological alterations of the gastrointestinal tract during aging, unfavorable socioeconomic conditions, in addition to the performance of hospital medical procedures, diagnostic or therapeutic, which affect food intake, poor monitoring of the patient’s nutritional status and lack of knowledge of standardized nutritional protocols [22,23,24,25]. Malnutrition at admission among hospitalized patients, is one of the most important negative predictors of the risk of a higher incidence of complications (e.g., in-hospital infections, pressure ulcers in bedridden patients), as well as responsible for a longer length of hospital stay (LOS), frequent rehospitalizations, poor response to treatments of the primary disease, and increased in-hospital mortality of patients [26,27,28,29].

Albumin, which is synthesized by the liver, has several key functions: it is the major serum binding protein, responsible for the transport of various substances, even drugs. It is also an acute-phase protein [30].

Hypoalbuminemia has been associated with increased short-term mortality, increased length of hospital stays, and increased occurrence of complications in hospitalized elderly patients [31,32,33,34,35,36,37,38].

Since albumin reflects hepatic protein synthesis and is reduced in catabolic states, serum albumin levels are a well-established surrogate marker in the measurement of malnutrition, being indicative of both hepatic synthesis, plasma distribution, and overall protein loss [26,27,28,29,39,40,41,42,43,44,45], particularly in elderly patients.

Findings from a previous large-scale cohort study have already highlighted the role of hypoalbuminemia as a risk factor for increased all-cause mortality among older adults, whether they live in the community or are hospitalized or institutionalized [46].

C-reactive protein (CRP) is an inexpensive, easy, and rapid inflammatory marker; it can correctly predict mortality. Measurement of CRP at hospital admission can help identify patients at increased risk of adverse outcomes, such as short- and long-term mortality [47,48,49,50]. Elevated CRP levels are known to be associated with an increased risk of all-cause mortality [51,52,53,54].

Both CRP and serum albumin (Alb) are useful markers for predicting morbidity and mortality in critically ill patients [47,55], as CRP is an effective marker of acute inflammation [56,57], whereas serum albumin (Alb) is an effective indicator of malnutrition status in critically ill patients [38,58]. The CRP/Alb ratio has recently been used as a predictor of prognosis in patients with severe sepsis or septic shock [59,60]; elevated CRP/Alb ratio at admission has been associated with a higher mortality rate in adult patients with sepsis. Even among intensive care or post-operative care patients and patients with severe burns, an elevated CRP/Alb ratio at admission has been independently and significantly associated with increased mortality rates, i.e., decreased 30-day survival [61].

CRP/ALB ratio has also been shown to be a useful prognostic marker in non-septic or otherwise non-infectious patients, such as patients with heart failure; a previous study has shown that an elevated CRP/ALB ratio is significantly associated with elevated in-hospital and out-of-hospital all-cause mortality in patients with acute and chronic heart failure. An elevated CRP/ALB ratio has also been associated with frequent and repeated hospital admissions, as well as an increased risk of developing severe heart failure [62]. The CRP/ALB ratio may, therefore, be a useful marker for assessment of critically ill patients, as it effectively reflects both inflammation and malnutrition [63,64]. An elevated CRP/ALB ratio at admission may be independently associated with an increased risk of 30-day mortality, as already demonstrated in a previous prospective study [65,66]. All the above-mentioned studies have shown that the elevated CRP/ALB ratio at the time of admission can be an independent predictor of mortality risk, simpler and more immediate than other prognostic assessment scales, such as APACHE II or Charlson Comorbidity Index. It is also true, however, that all these studies mainly assessed CRP/Alb in ICU, sepsis, or cancer patients.

Concerning patients not in critical conditions, the C-reactive protein/albumin ratio, together with the measurement of upper arm circumference and the assessment of impaired self-nutrition, are easily obtainable indicators of impaired energy and protein intake and poor clinical outcomes among hospitalized elderly [3]. This means that the CRP/ALB ratio, cost-effectively assessing both inflammation and malnutrition, can represent a useful additional parameter for the risk stratification of hospital mortality in elderly patients, regardless of diagnosis, even if it seems that this association is stronger in men than in women [67,68].

Unlike previous studies limited to intensive care units or disease-specific cohorts, we hypothesize that a higher CRP/Alb ratio at admission was independently associated with increased short-term (7-day) and early (30-day) in-hospital mortality in elderly patients, irrespective of the admission diagnosis.

The aim of our retrospective observational study on a cohort of elderly patients admitted to the Department of Internal Medicine and Aging of the Policlinico Riuniti in Foggia, Italy, was to evaluate the prognostic value of the CRP/Alb ratio as a predictor of the risk of hospital mortality at 7 and 30 days after admission.

## 2. Materials and Methods

### 2.1. Patients

A cohort of 3571 elderly patients admitted to the Department of Internal Medicine and Aging of the Policlinico Riuniti di Foggia, between 1 January 2019 and 31 December 2024, was screened. The exclusion criteria from the study were as follows: age less than 65 years at the time of admission; patients discharged against medical advice; patients transferred to other departments of the same hospital or other acute hospitals; patients discharged to nursing homes or rehabilitation institutions; patients with missing laboratory tests. The above patients were excluded to ensure a homogeneous sample of elderly patients with complete data for key biomarkers (CRP, albumin) at admission and definitive in-hospital outcomes. The final cohort consisted of 2780 subjects.

### 2.2. Methods

For all patients involved in the study, the following parameters were considered: serum C-reactive protein (CRP) and albumin values detected at the time of admission; length of stay (LOS); outcome of admission, i.e., discharge or death. All patients analyzed in our study were treated with medical therapy, either newly prescribed or confirming therapy already underway at home, total or partial, based on the clinical conditions of the patients. Comorbidity indices were not available in the hospital medical records and could not be derived retrospectively. The presence of a previous hospitalization in the thirty days preceding hospitalization was considered a severity marker. As for the causes of death, these were obtained from the hospital discharge forms present in the medical records, classified according to the ICD-9.

### 2.3. Statistics

The Kolmogorov–Smirnov test was performed to verify whether the data of the cohort under examination were normally distributed. Having verified that the examined data did not follow the normal distribution (*p* < 0.001), it was decided to perform the non-parametric Mann–Whitney U test for the comparison of means for independent samples, corrected with the exact Monte Carlo test; the non-parametric Spearman test was also performed to calculate correlations. The chi-square test, corrected with the Monte Carlo test, was performed to calculate the difference in frequencies between groups (males vs. females, deceased vs. non-deceased). Cohen’s d coefficient was performed to calculate the effect size on values expressed as mean and SD, assuming the following values of d: 0.2 = small effect; 0.5 = medium effect; 0.8 = large effect. Cohen’s h coefficient was performed to calculate the effect size on values expressed as percentages, i.e., deceased and non-deceased, or male and female, assuming the following values of phi, according to Cohen’s Guidelines: 0.2 = small effect; 0.5 = medium effect; 0.8 = large effect. The Phi coefficient was performed to calculate the effect size on the percentage values, i.e., deceased and non-deceased, stratified by sex, assuming the following values of phi: 0.1 = small effect; 0.3 = medium effect; 0.5 = large effect [69,70].

Both sensitivity and specificity, i.e., the predictive value of mortality of the CRP/Alb ratio, were calculated using ROC curve analysis; the optimal threshold value (optimal cut-off) was also identified. The Hazard Ratio (HR) for predicting mortality was calculated using Cox regression. Both the ROC curve analysis and the calculation of HR with Cox regression were performed after correction for sex.

Patient survival at 7 and 30 days from admission was estimated about the parameters examined, using Kaplan–Meier analysis, obtaining the relative survival curves. The presence of significant differences between the two survival curves, both at 7 and 30 days, was then analyzed using the Log-rank test, stratified by age at admission. We also performed sensitivity analyses, both including all subjects and after excluding those with a LOS equal to or greater than seven days.

The statistical packages IBM SPSS version 25 (Armonk, NY, USA) and STATA SE 14.2 (College Station, TX, USA) were used. *p* values lower than 0.05 were considered statistically significant.

## 3. Results

The features of the cohort examined are presented in Table 1. We found that, in our cohort, women were more represented than men (*p* = 0.005). As expected, women were older than men (*p* < 0.001). We did not find any statistically significant differences between men and women concerning both LOS and serum values of Albumin. We found higher CRP values (*p* = 0.006) and higher CRP/Alb ratio values among men (*p* = 0.006) than among women.

Aware that the simple definition of patients over 65 years of age might be too broad, we stratified the cohort by age subgroups [71,72], i.e., “young old” (65–74 years old), “old” (75–84 years old), “very old” (85 + years old), as presented in Table 2. Men were more represented than women among the young elderly (*p* < 0.001), while women were more represented than men among the very old (*p* < 0.001). No statistically significant difference was found between men and women among the elderly (*p* = 0.705).

No further analyses were conducted in subgroups by age category in this study but are planned in future work.

Correlation analysis, corrected for sex, highlighted, as expected, a statistically significant direct relationship between both CRP and CRP/Alb ratio and LOS (*p* < 0.001); a statistically significant inverse relationship was highlighted between serum albumin values and LOS (*p* < 0.001); no statistically significant correlation was observed between the age of the patients at admission and LOS (*p* = 0.441). The results of the correlation analysis are shown in Table 3.

In total, 444 patients died during their hospital stay. The main causes of death are reported in Table 4. According to previous studies, and according to data collected by the Italian National Cause of Death Register, managed by the Italian National Institute of Statistics (ISTAT) [73,74], severe sepsis is the most frequent cause of in-hospital death. We did not perform any inferential statistical comparisons between causes of death, as these were reported primarily for descriptive purposes.

Deceased subjects were older than non-deceased subjects (*p* < 0.001); no significant differences were found between males and females for the proportion of deaths (*p* = 0.276) or for LOS (*p* = 0.368). Compared with non-deceased subjects, serum albumin values were lower, and CRP values were higher among deceased subjects; consequently, the CRP/Alb ratio was significantly higher among deceased subjects (*p* < 0.001) compared with non-deceased patients. Characteristics of deceased and non-deceased patients and serum albumin and CRP values and the Alb/CRP ratio (all expressed as mean and SD) are reported in Table 5.

**Figure 1:**
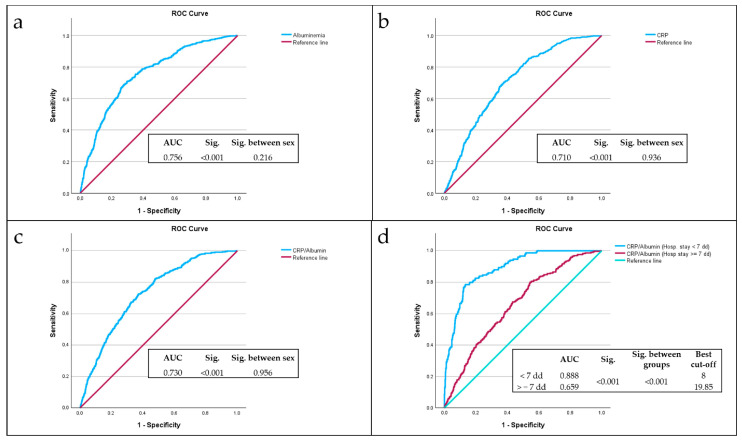
Analysis of ROC curves of Albumin (**a**), CRP (**b**), CRP/Alb ratio (**c**), CRP/Alb ratio among LOS less than seven days or equal to or greater than seven days (**d**) as predictors of mortality, compared with the reference line (red line).

ROC curve analysis highlighted the significant predictive role of albumin (Figure 1a), CRP (Figure 1b), and the CRP/Alb ratio (Figure 1c) in mortality. From the comparison of the area under the curve (AUC) values, the most accurate predictor of mortality was albumin, with an AUC of 0.756, with no difference between sexes (*p* = 0.216); CRP showed an AUC of 0.710 with no difference between sexes (*p* = 0.936). The CRP/Alb ratio showed an AUC of 0.730, with no difference between sexes (*p* = 0.956). After dividing the sample according to hospital stay days, i.e., according to hospital stay days less than seven days or equal to or greater than seven days,

The CRP/Alb ratio demonstrated excellent discriminative ability among subjects with hospital stays less than seven days, with an AUC of 0.888 and the best cut-off value of 8 (Figure 1d), then subjects with hospital stays equal to or greater than seven days (*p* < 0.001).

The best cut-off value of CRP/Alb ratio of 8 was identified via ROC analysis as the point of highest combined sensitivity and specificity for 7-day mortality, with a sensitivity of 89%, a specificity of 66% and a Youden Index of 0.543.

Sensitivity analyses performed subsequently showed that the CRP/Alb ratio greater than 8 had a sensitivity of 85.8% in predicting mortality for all patients, while it had a sensitivity of 89.7% in predicting mortality for patients with a LOS of less than seven days.

**Figure 2:**
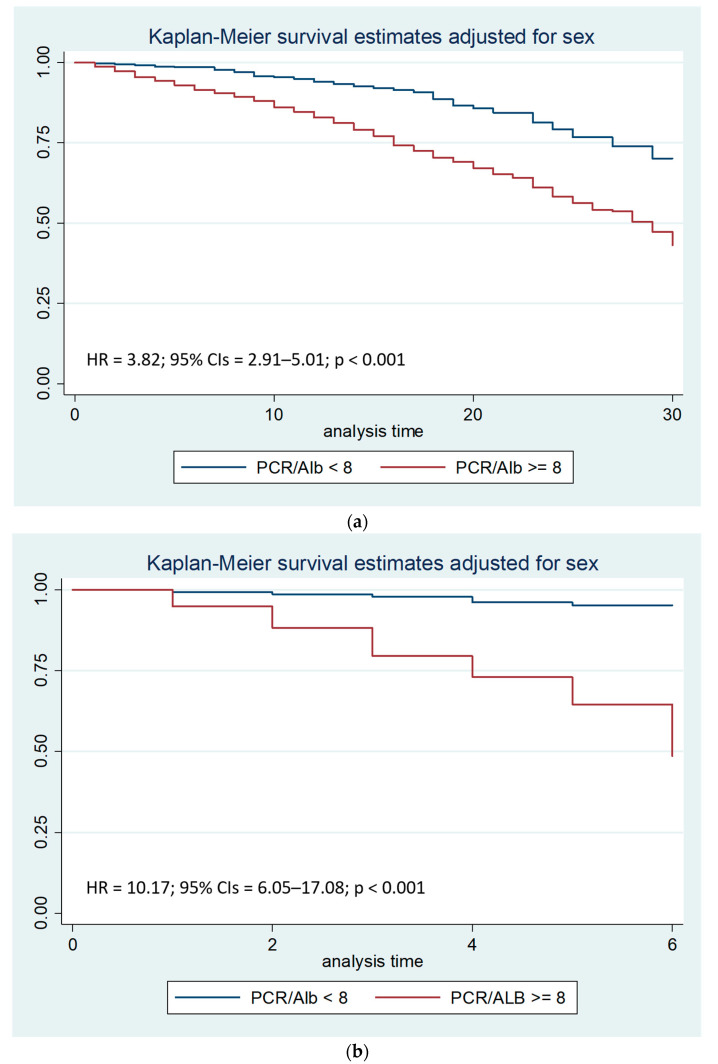
(**a**). Kaplan–Meier survival estimates adjusted for sex stratified by PCR/Alb ratio < 8 vs. PCR/Alb ratio >= 8 (chi-square 66.41; *p* < 0.001; 95% CIs: 95% Confidence Intervals). (**b**). Kaplan–Meier survival estimates adjusted for sex stratified by PCR/Alb ratio < 8 vs. PCR/Alb ratio >= 8 during the first seven days of hospitalization (chi-square 124.14; *p* < 0.001; 95% CIs: 95% Confidence Intervals).

Cox regression analysis with the Breslow method, adjusted for sex and hospital stay days less than, equal to, or greater than seven days, highlighted that a CRP/Alb ratio greater than 8 is an independent risk factor for mortality during the first thirty days of hospitalization, with a Hazard Ratio (HR) of 3.82 (Figure 2a). In particular, a CRP/Alb ratio greater than 8 is a significant risk factor for mortality during the first seven days of hospitalization, with a HR of 10.17 (Figure 2b).

Survival analysis to compare the two Kaplan-Meier curves, performed with the Log-rank test, after stratifying the sample by sex, showed that the PCR/Alb ratio higher than 8 was associated with a reduction in survival (*p* < 0.001), both after thirty days and after seven days from hospitalization.

The hospitalization involving 370 patients was a rehospitalization, or a return to the hospital within thirty days of a previous discharge. In particular, for these patients, a PCR/Alb ratio higher than 8 was associated with a significant increase in the risk of mortality (*p* < 0.001) during the first seven days of hospitalization, with an Odds Ratio (OR) of 5.13, as shown in Table 6.

## 4. Discussion

The present study demonstrates that the C-reactive protein/albumin (CRP/Alb) ratio is a significant independent predictor of short-term mortality among hospitalized elderly patients, particularly in the first days following admission. The predictive value of this readily available biomarker is especially relevant in the geriatric setting, whereas systemic inflammation and malnutrition, two key components captured by CRP and albumin, frequently coexist and contribute to adverse outcomes.

While our findings are consistent with prior studies highlighting the prognostic relevance of the CRP/Alb ratio in critically ill or elderly populations [38,47,55,56,57,58,59,60,63,64], they also expand its applicability to a broader inpatient elderly cohort, regardless of admission diagnosis. Importantly, the association held even in patients with short hospital stays, with the ratio achieving its highest predictive accuracy (AUC = 0.888) in those hospitalized for under seven days. This suggests that the CRP/Alb ratio may be particularly valuable for identifying patients at risk of early mortality, an insight with direct implications for acute care management.

Nevertheless, these findings should be contextualized within the broader literature. Some studies in general or outpatient populations have found only limited predictive value for the CRP/Alb ratio, particularly when used outside intensive care or in less frail cohorts. However, these findings should be contextualized with the broader literature. Some studies in general or outpatient populations have found only limited predictive value for the CRP/Alb ratio, particularly when used outside of the ICU or in less frail cohorts. These discrepancies may reflect population differences, as the ratio likely works best in settings with high initial risks, such as acute care unit or ICU admissions.

Furthermore, while both CRP and albumin individually predict mortality, their combination in a ratio may offer an additive effect by jointly reflecting inflammatory burden and nutritional reserve. This dual-pathway insight may explain the greater discriminatory ability observed in our high-risk cohort. Moreover, while both CRP and albumin individually predict mortality, their combination in a ratio may offer an additive effect by jointly reflecting inflammatory burden and nutritional reserve. This dual-pathway insight could explain the enhanced discriminative ability observed in our high-risk cohort.

From a clinical perspective, a CRP/Alb ratio > 8 at admission may serve as a useful red flag. Patients exceeding this threshold could be prioritized for early geriatric assessment, nutritional intervention, and closer monitoring during hospitalization. Furthermore, such patients, particularly those admitted within 30 days of a previous hospital discharge, may benefit from discharge planning strategies focused on nutritional support and inflammatory control. However, given the observational nature of the study and the lack of adjustment for key clinical variables (e.g., comorbidities, medications, functional status), the CRP/Alb ratio should inform, but not solely dictate, clinical decisions.

These findings reinforce a growing recognition that malnutrition and inflammation are not merely background factors but central determinants of prognosis in older adults. The CRP/Alb ratio captures the bidirectional interplay between these two domains: systemic inflammation drives protein catabolism and reduces albumin synthesis, while malnutrition impairs immune competence, fueling inflammation. As such, this ratio offers a more integrative risk marker than CRP or albumin alone.

An additional strength of our study is its large, homogeneous elderly cohort (*n* = 2780), enabling robust mortality estimates. The exclusion of younger patients minimized confounding by age-related resilience, while the inclusion of early mortality and rehospitalization outcomes enhances clinical relevance. Still, limitations must be acknowledged. The retrospective design precludes causal inference, and only baseline laboratory data were available. Serial measurements of CRP and albumin would have allowed for dynamic monitoring of response to interventions or disease progression. Furthermore, the lack of comorbidity indices such as the Charlson Comorbidity Index or APACHE II reduces our ability to fully contextualize the predictive value of CRP/Alb in comparison with established clinical risk scores.

Despite these caveats, our results support the incorporation of the CRP/Alb ratio into early risk stratification protocols for elderly inpatients. In resource-limited settings especially, this inexpensive, routinely available biomarker could aid in identifying high-risk individuals who may benefit from tailored interventions. Moreover, our findings align with existing evidence advocating for routine nutritional screening upon admission, as per ESPEN guidelines [4], and suggest that integrating inflammatory biomarkers may enhance these assessments.

Finally, although this study did not find a statistically significant sex-based difference in the predictive value of CRP/Alb, emerging literature suggests that inflammatory responses and outcomes may differ by sex [68]. Future research with sex-stratified analyses could clarify whether CRP/Alb thresholds should be tailored accordingly.

In conclusion, our study further highlights the role of the CRP/Alb ratio as a simple yet powerful prognostic tool for short-term mortality in hospitalized elderly patients. A CRP/Alb ratio > 8 identifies patients at particularly high risk of early death and could be integrated into routine clinical workflows to prompt early, targeted interventions. Prospective validation in diverse settings, ideally with serial measurements and comprehensive clinical data, is warranted to confirm its utility and refine its application in geriatric care.

## 5. Conclusions

This retrospective study on a large and homogeneous cohort of elderly inpatients supports the prognostic value of the CRP/albumin (CRP/Alb) ratio in predicting early in-hospital mortality. By capturing the combined effects of systemic inflammation and malnutrition, the CRP/Alb ratio emerged as an independent risk factor for mortality, with particularly strong predictive performance within the first seven days of admission. A threshold value of 8 was identified as a potential discriminator of high risk.

The study’s strengths include its large sample size and focus on patients aged 65 and older, enhancing its relevance to geriatric care. However, important limitations must be acknowledged. Its retrospective design, reliance solely on admission laboratory values, and lack of adjustment for key clinical covariates such as comorbidities and functional status limit the ability to draw causal conclusions or support clinical implementation.

While the CRP/Alb ratio shows promise as a simple, cost-effective early warning tool for identifying elderly patients at elevated mortality risk, these findings should be interpreted as exploratory. Prospective studies incorporating serial biomarker measurements, clinical severity indices, and functional assessments are essential to validate the prognostic utility of the CRP/Alb ratio. Only then can its role in routine risk stratification and clinical decision-making in geriatric care be confidently defined.

## Figures and Tables

**Table 1:** Clinical features of patients, stratified by sex. Values are expressed as number (%) or as mean ± SD.

	**Male**	**Female**	**Effect Size** **Cohen’s h**	**Sig.** **(99% CIs)**
Subjects N (%)	1316 (47.3%)	1464 (52.7%)	0.11	0.004 (0.001–0.008)
	**Male**	**Female**	**Effect Size** **Cohen’s d**	**Sig.** **(99% CIs)**
Age at hospitalization (Mean ± SD)	79 ± 8	82 ± 8	0.32	<0.001 (0.000–0.002)
LOS (Mean ± SD)	11 ± 7	11 ± 7	0.01	0.947 (0.936–0.958)
Albuminemia (Mean ± SD)	3.05 ± 0.6	3.03 ± 0.6	0.02	0.751 (0.730–0.772)
CRP (Mean ± SD)	80.1 ± 89	73.2 ± 88	0.08	<0.001 (0.000–0.002)
CRP/Alb ratio (Mean ± SD)	31.3 ± 38	29.2 ± 40	0.05	0.476 (0.452–0.501)

**Table 2:** Features of patients, stratified by age groups. Values are expressed as number (%).

	Male	Female	Effect Size Cohen’s h	Sig. (99% CIs)
Young old N (%) (65–74 years old)	382 (56.9%)	289 (43.1%)	0.28	<0.001 (0.000–0.002)
Old N (%) (75–84 years old)	551 (49.4%)	565 (50.6%)	0.02	0.705 (0.682–0.727)
Very Old N (%) (85 + years old)	383 (38.6%)	610 (61.4%)	0.46	<0.001 (0.000–0.002)

**Table 3:** Correlation between LOS and age at admission, serum albumin, CRP, and CRP/Alb after correcting by sex.

		Length of Stay
Age at admission	Partial correlation	−0.015
	Significance (2-tailed)	0.441
CRP	Partial correlation	0.148
	Significance (2-tailed)	<0.001
Albuminemia	Partial correlation	−0.189
	Significance (2-tailed)	<0.001
CRP/Alb ratio	Partial correlation	0.150
	Significance (2-tailed)	<0.001

**Table 4:** Main causes of death among study subjects. Values are expressed as number (%).

	Deceased
Severe Sepsis N (%)	221 (49.8%)
Pulmonary edema and respiratory failure N (%)	44 (9.9%)
Any respiratory infection and inflammation with complications N (%)	43 (9.7%)
Pleural effusion with complications N (%)	19 (4.3%)
Heart failure and shock N (%)	19 (4.3%)
Malignant neoplasms of digestive system with complications N (%)	9 (2.0%)
Any infectious disease N (%)	9 (2.0%)
Severe renal failure N (%)	8 (1.8%)
All other causes N (%)	72 (16.2%)
**Total number of deaths N (%)**	444 (100.0%)

**Table 5:** Clinical features of patients, stratified by deceased and not deceased. Values are expressed as number (%) or as mean ± SD.

	**Deceased**	**Non-Deceased**	**Effect Size** **Cohen’s h**	**Sig.** **(99% CIs)**
Subjects N (%)	444 (16%)	2336 (84%)	1.50	<0.001 (0.000–0.002)
	**Deceased**	**Non-Deceased**	**Effect Size** **Phi Coefficient**	**Sig.**
Male N (%)	221 (49.8%)	1095 (46.9%)	0.02	0.276
Female N (%)	223 (50.2%)	1241 (53.1%)		
	**Deceased**	**Non-Deceased**	**Effect Size** **Cohen’s d**	**Sig.** **(99% CIs)**
Age at hospitalization (Mean ± SD)	84 ± 8	80 ± 8	0.411	<0.001 (0.000–0.002)
LOS (Mean ± SD)	12 ± 10	11 ± 7	0.098	0.003 (0.000–0.005)
Albuminemia (Mean ± SD)	2.6 ± 0.6	3.1 ± 0.6	−0.955	<0.001 (0.000–0.002)
CRP (Mean ± SD)	123.6 ± 95.9	67.5 ± 84.1	0.651	<0.001 (0.000–0.002)
CRP/Alb ratio (Mean ± SD)	54.8 ± 47.8	25.6 ± 35.8	0.771	<0.001 (0.000–0.002)

**Table 6:** Relationship between PCR/Alb ratio more than 8, LOS, and risk of death, among re-hospitalized patients.

	OR	95% CIs	Sig.
LOS >= 7 days	5.13	2.38–12.62	<0.001
LOS < 7 days	15	3.99–81.82	

## Data Availability

Data are unavailable due to privacy restrictions.
